# Nerve Growth Factor Neutralization Promotes Oligodendrogenesis by Increasing miR-219a-5p Levels

**DOI:** 10.3390/cells10020405

**Published:** 2021-02-16

**Authors:** Rossella Brandi, Marietta Fabiano, Corinna Giorgi, Ivan Arisi, Federico La Regina, Francesca Malerba, Sabrina Turturro, Andrea Ennio Storti, Flavia Ricevuti, Susanna Amadio, Cinzia Volontè, Simona Capsoni, Raffaella Scardigli, Mara D’Onofrio, Antonino Cattaneo

**Affiliations:** 1 European Brain Research Institute (EBRI) “Rita Levi-Montalcini”, Viale Regina Elena, 295, 00161 Rome, Italy; r.brandi@ebri.it (R.B.); mariettafabiano89@gmail.com (M.F.); corinna.giorgi@cnr.it (C.G.); i.arisi@ebri.it (I.A.); f.laregina@ebri.it (F.L.R.); f.malerba@ebri.it (F.M.); sabrina.turturro55@gmail.com (S.T.); andrea.ennio.storti@gmail.com (A.E.S.); fricevuti@gmail.com (F.R.); 2 CNR, Institute of Molecular Biology and Pathology (IBPM), P.le Aldo Moro, 5, 00185 Rome, Italy; 3 CNR, Institute of Translational Pharmacology (IFT), Via del Fosso del Cavaliere 100, 00131 Rome, Italy; 4 IRCCS Fondazione Santa Lucia, Preclinical Neuroscience, Via del Fosso di Fiorano 65, 00143 Rome, Italy; s.amadio@hsantalucia.it (S.A.); cinzia.volonte@cnr.it (C.V.); 5 CNR, Institute for Systems Analysis and Computer Science, Via Dei Taurini 19, 00185 Rome, Italy; 6 Bio@SNS, Scuola Normale Superiore, 56124 Pisa, Italy; simona.capsoni@sns.it; 7 Institute of Physiology, Department of Neuroscience and Rehabilitation University of Ferrara, 44121 Ferrara, Italy

**Keywords:** Nerve growth factor (NGF), miR-219a-5p, oligodendrogenesis, myelin, demyelinating diseases, microRNAs

## Abstract

In the brain, the neurotrophin Nerve growth factor (NGF) regulates not only neuronal survival and differentiation, but also glial and microglial functions and neuroinflammation. NGF is known to regulate oligodendrogenesis, reducing myelination in the central nervous system (CNS). In this study, we found that NGF controls oligodendrogenesis by modulating the levels of miR-219a-5p, a well-known positive regulator of oligodendrocyte differentiation. We exploited an NGF-deprivation mouse model, the AD11 mice, in which the postnatal expression of an anti-NGF antibody leads to NGF neutralization and progressive neurodegeneration. Notably, we found that these mice also display increased myelination. A microRNA profiling of AD11 brain samples and qRT-PCR analyses revealed that NGF deprivation leads to an increase of miR-219a-5p levels in hippocampus and cortex and a corresponding down-regulation of its predicted targets. Neurospheres isolated from the hippocampus of AD11 mice give rise to more oligodendrocytes and this process is dependent on miR-219a-5p, as shown by decoy-mediated inhibition of this microRNA. Moreover, treatment of AD11 neurospheres with NGF inhibits miR-219a-5p up-regulation and, consequently, oligodendrocyte differentiation, while anti-NGF treatment of wild type (WT) oligodendrocyte progenitors increases miR-219a-5p expression and the number of mature cells. Overall, this study indicates that NGF inhibits oligodendrogenesis and myelination by down-regulating miR-219a-5p levels, suggesting a novel molecular circuitry that can be exploited for the discovery of new effectors for remyelination in human demyelinating diseases, such as Multiple Sclerosis.

## 1. Introduction

In the central nervous system (CNS), oligodendrocytes (OLs) are responsible for generating myelin sheaths, insulating axons, and promoting efficient action potential propagation. During prenatal development, OL precursor cells (OPCs) are generated in multiple waves and migrate to different brain areas, contributing to generate mature, myelinating OLs for the entire CNS [1]. This process can continue in the adult, capitalizing on a small fraction of quiescent OPCs, which, upon myelin damage, can be induced to proliferate, migrate to the lesion site, and finally differentiate into mature OLs, replenishing OL loss [2,3,4,5]. Unfortunately, the efficacy of this regenerative process varies greatly with age and with the nature of the lesion, often leading to incomplete remyelination, particularly in chronic inflammatory demyelinating diseases, such as Multiple Sclerosis (MS) [6,7,8]. The molecular mechanisms underlying OPC reactivation and differentiation have been extensively studied [9], revealing a complex orchestra of opposing signals, intrinsic and extrinsic factors, including cytokines, growth factors, hormones, and neurotrophins (NTs) [2]. NTs are involved in a plethora of physiological processes and they are endogenously released by many cell types, including neurons, oligodendrocytes, and Schwann cells (SC) [10]. NTs are synthesized as proneurotrophins, which are then proteolytically cleaved into the mature NT. While proneurotrophins primarily exert their effects upon binding the pan-neurotrophin receptor p75^NTR^, and its sortilin (Vps10p domain) co-receptor, mature NTs bind p75^NTR^ with low affinity and the tropomyosin-related kinase receptor with high affinity (TrkA for Nerve growth factor (NGF), TrkB for Brain-derived growth factor (BDNF), and TrkC for Neurotrophin-3 (NT-3)) [11]. The NTs NGF, BDNF, and NT-3 all participate in modulating myelin formation. In both the CNS and in the peripheral nervous system (PNS), they mediate intercellular communication between nerve and glia cells, regulating axonal signals that control myelination or affecting glia cell function and maturation directly [12,13]. NT-3 and BDNF promote both peripheral and central myelination [14,15,16]. However, while NGF acts as positive effector on PNS Schwann cells (SC), it plays the opposite role in the CNS, reducing oligodendrocyte differentiation and myelination [17,18,19,20]. In particular, the NGF-mediated inhibition of OPC differentiation occurs via p75^NTR^ signaling [17,20]. The opposite effects of NGF may be due to the differential expression of NGF receptors (TrkA, p75^NTR^ and sortilin) in the two cell types (SC versus OL) at different stages of maturation [17,19].

Despite this important evidence, the role of NGF in oligodendrogenesis and myelination is still poorly defined, as are the underlying mechanisms involved.

In this study, to better understand the molecular pathway through which NGF modulates myelination, we investigated NGF downstream targets that are relevant to oligodendrogenesis, focusing our attention on microRNAs (miRNAs). Indeed, miRNAs are emerging as key molecular effectors of oligodendrogenesis and attractive therapeutic targets [21,22,23,24,25]. To identify candidate miRNAs that may mediate the role exerted by NGF in oligodendrogenesis, we took advantage of the AD11 mouse model of NGF deprivation [26]. These transgenic mice express a neutralizing anti-NGF antibody postnatally and develop a progressive neurodegeneration, which is characterized by severe cholinergic and behavioral deficits and by significant impairments of cortical and hippocampal synaptic plasticity [27,28,29,30]. We found that the neutralization of NGF is accompanied by an increase in myelinating OLs, confirming its role as a negative regulator of oligodendrogenesis. A microarray analysis of AD11 brain tissue revealed a significant dysregulation of several miRNAs. Among them, miR-219a-5p (hereafter referred to as miR-219) emerged as one of the most up-regulated miRNAs, as also confirmed in AD11-derived OPC progenitors. Notably, miR-219 is a well characterized positive regulator of oligodendrocyte differentiation and myelin repair [31,32,33,34]. These data suggest that NGF may affect myelination by inhibiting the miR-219 levels. Indeed, while NGF-deprived neurospheres gave rise to more oligodendrocytes, this enhancement was lost upon decoy-mediated depletion of miR-219. Further, the NGF treatment of AD11 neurospheres inhibited miR-219 up-regulation and, consequently, OL differentiation. Conversely, the treatment of WT progenitors with anti-NGF antibodies increased miR-219 expression and the number of mature oligodendrocytes. Altogether, our data indicates that NGF inhibits oligodendrogenesis by down-regulating the expression miR-219, unveiling a new molecular cascade that modulates myelination.

## 2. Materials and Methods

### 2.1. Anti-NGF AD11 Mouse Model

This study includes AD11 transgenic mice, which obtained ethical approval by the Italian Health Ministry (code n. 1214/2015-PR). AD11 transgenic mice [26] express a recombinant version of the monoclonal antibody mAb αD11 that specifically recognizes and neutralizes NGF [35,36]. As in previous studies [28], AD11-VH mice were used as transgenic control mice that are consistently negative with respect to all of the neurodegeneration markers. Each AD11 and AD11-VH control mouse was individually tested by transgene genotyping (for VH and VK transgenes). The mRNA for the VH and VK antibody chains was also determined in each AD11 and AD11-VH mouse, by Real-Time qRT-PCR. Mice were kept under a 12 h dark to light cycle, with food and water *ad libitum*. All experiments with transgenic and control mice were conducted according to national and international laws for laboratory animal welfare and experimentation (Italian Legislative Decree n°26/2014 and European Union Directive n°2010/63/UE).

### 2.2. RNA Isolation, Amplification and Labeling

Hippocampi of the right hemisphere were dissected from the hippocampus of one month-old AD11 and control mice and total RNA was isolated using Trizol (Invitrogen, San Diego, CA) and DNAse treated by Qiagen columns. Quality and integrity of each sample was controlled for using the Agilent BioAnalyzer 2100 (Agilent RNA 6000 nano kit). Only samples with a RNA Integrity Number (RIN) index higher than 8.0 were selected. All the experimental steps involving the labeling, hybridization and washings of the samples were done following the Agilent protocol (http://chem.agilent.com (accessed on 22 February 2021), miRNA Microarray System with miRNA Complete Labeling and Hyb Kit protocol, version 3.1.1, Agilent Technologies, Santa Clara, CA, USA). The Small RNA kit was used for the separation and quantification of miRNA. Labeled miRNAs were obtained from 100 ng of total RNA through the ligation of a 5′-cytidine bisphosphate-Cy3 (pCp-Cy3, Agilent Technologies) group at the 3′-end of each miRNA. We had previously treated total RNA with alkaline phosphatase (TaKaRa Bio Inc, Kusatsu, Japan) at 37 °C for 30 min. to enhance the T4 RNA-ligase (Ambion, ThermoFisher, Waltham, MA, USA) efficiency.

### 2.3. Microarray Analysis

Labeled miRNAs were purified on chromatography columns (Micro Biospin 6, Biorad Laboratories, Hercules, CA, USA) and then hybridized on a microarray. Each slide contains eight identical microarrays containing probes for 567 mouse and 73 mouse viral miRNAs. Hybridizations were performed at 55 °C for 20 h in a rotating oven. The hybridized microarrays were disassembled at room temperature in Agilent Gene Expression Wash Buffer 1. After the disassembly, the microarrays were washed in Gene expression Buffer 1 for five minutes at room temperature, followed by washing with Gene Expression Wash Buffer 2 for five minutes at 37 °C. The microarrays were then treated with Acetonitril for five minutes at room temperature. Post-hybridization image acquisition was performed using the Agilent scanner G2564B, which was equipped with two lasers (532 nm and 635 nm) and a 48 slides auto-sampler carousel. Data extraction from the images was accomplished by Agilent Feature Extraction 9.5 software using the standard Agilent one-color miRNA expression extraction protocol. Data analyses were performed using Agilent GeneSpring GX (version 11, Agilent Technologies, Santa Clara, CA, USA), MultiExperimentViewer (TIGR) and Microsoft Excel.

### 2.4. Microarray Data Analysis

Differential expressed miRNAs have been selected from microarray data using a combination of two thresholds: fold change >2.0 and T-test *p*-value <0.05. mRNA targets have been predicted using TargetScan ver 6.2 [37], with threshold Score of <= −0.1. The functional analysis of gene lists was obtained using DAVID [38,39]. The microarray gene expression data that were discussed in this publication have been deposited in NCBI’s Gene Expression Omnibus (GEO) [40], and they are accessible and publicly available through GEO Series accession number GSE162675.

### 2.5. RNA Isolation and Reverse Transcription-PCR

RNA was isolated from AD4 and wild type (WT) neurosphere cultures or from different tissues from AD11 and WT mice. Briefly, the neurospheres or brain areas were lysed with Trizol (Invitrogen, San Diego, CA, USA), DNAse treated, and purified with Qiagen columns (QIAGEN Sciences, MD, USA). RNA quantity was determined on a NanoDrop UV-VIS. Each sample was then quality checked for integrity using the Agilent BioAnalyzer 2100 (Agilent G2938C, RNA 6000 nano kit).

Taqman miR219a-5p analysis (Applied Biosystems Assay ID: 000522) was performed on 10ng of RNA, following the manufacturer’s protocol (Applied Biosystems, ThermoFisher, Waltham, MA, USA), and then normalized to U6 snRNA (Applied Biosystems Assay ID: 001973). Real-Time qRT-PCR, which was used to detect the expression levels of mouse target-mRNAs, was performed with an ABI StepOnePlus Real Time PCR system (following manufacturer’s instructions) and normalized to Ppia mRNA levels. qRT-PCR was performed on an Applied Biosystems 7900HT thermal cycler. Primers were as follows: Camk2a (forward 5′-tggagactttgagtcctacacg-3′ and reverse 5′-ccgggaccacaggttttca-3′); Cank2g (forward 5′-gagactgaggcccgatttct-3′ and reverse 5′-tgtcaggaaaaccaacagga-3′); Fgfr2 (forward 5′-accctctctgacccaccat-3′ and reverse 5′-cacctgtctgccttgagtcc-3′); Hes5 (forward 5′-tacctgaaacacagcaaagccttc-3′ and reverse 5′-taaagcagcttcatctgcgtgtcg-3′); Neurod1 (forward 5′-cacgtcttccacgtcaagc-3′ and reverse 5′-gtcagttagggggctttcaa-3′); Pdgfa (forward 5′-ttggatgacattttcgtcgat-3′ and reverse 5′-cgtctcgctgtcccaact-3′); Pdgfra (forward 5′-acacgcgccaacttgtact-3′ and reverse 5′-aacccatcctcggttaagagtt-3′); Sox6 (forward 5′-tcagagcaatcaccacaccagaca-3′ and reverse 5′-aaggttgaatgtcagggcaaaggc-3′); and, Zfp238 (forward 5′-gatggtccccagtgagagaa-3′ and reverse 5′-tctcaagcaaaccttcacagag-3′). The data were analyzed using the ddCT method.

### 2.6. Brain Dissection and Tissue Processing

One month-old AD11 and control mice were intracardially perfused with 4% paraformaldehyde, whole brains extracted, and fixation continued in 4% paraformaldehyde overnight at 4 °C. After cryoprotection in 30% sucrose, the brains were cryosectioned at 40 µm of thickness, and coronal sections encompassing cortex, corpus callosum, and septum were analyzed by immunohistochemistry.

### 2.7. Neural Stem Cell Cultures

Neurospheres were derived from the hippocampi of WT animals and AD11 mice (AD4 neurospheres), as previously described [41]. The cells were cultured in Dulbecco’s modified Eagle’s medium (DMEM)/F12 medium that was supplemented with B27 (Invitrogen, San Diego, CA), epidermal growth factor (EGF), and basic fibroblast growth factor (bFGF) (20 and 10 ng/mL, respectively; Peprotech, London, UK; NSC growth medium) in a humidified incubator at 37 °C in 5% CO_2_. The growth factors were replenished weekly. In order to induce oligodendrocytes differentiation, neurospheres were mechanically dissociated into single cells upon accutase (Sigma, St. Louis, MO, USA) treatment and plated onto matrigel-coated glass coverslips (12 mm diameter) at 5 × 10^5^ mL^−1^ cell density in OPC differentiation medium (growth medium without EGF and FGF, containing 1% horse serum and 10 ng/mL of CNTF). Five or ten days after plating, the cultures were fixed in 4% PFA and then processed for immunocytochemistry with O4 and MBP antibodies.

### 2.8. Oligodendrocytes Primary Cultures

Purified cultures of oligodendrocytes were prepared from forebrain of postnatal day 1–2 Wistar rats, as described [42]. The cells were then induced to differentiate into mature oligodendrocytes when the basal chemically defined medium (DMEM, 4 mM L-glutamine, 1 mM sodium pyruvate, 0.1% bovine serum albumin (BSA), 50 µg/mL apo-transferrin, 5 µg/mL insulin, 30 nM sodium selenite, 10 nM D-biotin, and 10 nM hydrocortisone) was added with 15 nM triiodothyronine, 10 ng/mL ciliary neurotrophic factor (CNTF) and 0.05 mg 10 mL^−1^ Nacetyl-L-cysteine (NAC), for 4–7 days. The cultures were fixed in 4% PFA for 10 min and processed for immunocytochemistry with anti-oligodedrocyte marker O4 (O4) and anti-myelin basic protein (MBP) antibodies to assess OL differentiation.

### 2.9. NGF and Anti-NGF αD11 Antibody Treatment

For NGF and anti-NGF treatments, in-house produced recombinant mouse NGF at 50 ng/mL [36], or mAb αD11 at 100 ng/mL [43], were added to neurospheres or primary OPCs prior to (five days) and during (5 to 10 days) OL differentiation. Proliferating and differentiating media (for neurospheres and OPCs) were replenished every other day adding fresh NGF or mAb αD11.

### 2.10. Immunofluorescence on Brain Section and OLs

The immunohistochemistry of AD11 and WT brains was performed on 40-µm serial free-floating sections. Upon fixation, sections or cells were permeabilized in 0.3% or 0.1% Triton X-100, respectively, in PBS, and then incubated with the antibody of interests (anti-O4: MerkMillipore, clone 81, MAB345, 1:50; anti-myelin basic protein (MBP): Santa Cruz Biotechnology, CA, USA; sc-13914, 1:100). The total number of cells in each field was determined by counterstaining cell nuclei with 4,6-diamidine- 2-phenylindole dihydrochloride (DAPI; Sigma–Aldrich; 50 mg/mL in PBS for 15 min. at RT). Immunostained sections and cells were mounted in Aqua-Poly/Mount (Polysciences, Inc., Warrington, PA, USA, http://www.polysciences.com (accessed on 22 February 2021)) and then analyzed by confocal microscopy, using a TCS SP5 microscope (Leica Microsystem, Wetzlar, Germany). Z-stacks images were captured at 1 µm intervals with a 40× or 60× objectives and a pinhole of 1.0 Airy unit. Analyses were performed in sequential scanning mode to rule out bleed-through between channels. Fluorescence intensity quantification was performed with ImageJ software. Quantification of number and length of branches in O4+ OL was performed with ImageJ software. Branch length was reported in µm as the maximal distance measured from the OL soma.

### 2.11. Design, Production and Transduction of Lentiviral Vectors

Lentiviral plasmids pLB_miR-219 and pLB_miR-ct were designed to obtain an overexpression of miR-219 or of a non-targeting control miR respectively. Cloning and production procedures were performed, as previously described [44]. The plasmids were cloned inserting the pre-miR-219 (or pre-miR cntr) dsDNA sequences into the HpaI e XhoI sites of the pLB lentiviral plasmid, under the control of the U6 promoter. These plasmids also encode for GFP, to allow for the identification of transduced cells. The sense strand of the cloned fragments is as follows:

pre-miR-219: TGTGATTGTCCAAACGCAATTCTTTCAAGAGAAGAATTGCGTTTGGACAATCATTTTTC

pre-miR-cntr: TGCAACAAGATGAAGAGCACCAACTCGAGTTGGTGCTCTTCATCTTGTTGCTTTTTC

To produce third generation lentiviral particles LV_miR-219GFP and LV_miR-ct_GFP, Hek293T cells were Ca-PO^4^ transfected with the corresponding lentiviral plasmid (either pLB_miR-219 or pLB_miR-ct) and three packaging plasmids. At 48 and 72 h from transfection, the growth media containing lentiviral particles was filtered (0.4 mm pore size) and subjected to ultracentrifugation at 26,000 rpm for 1.5 h in a SW40Ti rotor. Pelleted virions were resuspended in PBS, aliquoted, and stored at −80 °C. Viral Titer (in transducing units: TU) was tested for each preparation, and it varied between 1.5 × 10^8^ TU/mL and 1.2 × 10^9^ TU/mL. Anti-miR-219 tough decoy inhibitor (decoy_miR-219) and the non-targeting control lentiviruses were purchased from Sigma Aldrich (cat. decoy_miR-219: MLTUD0339; cat. decoy_miR-c: HLTUD001C). All lentiviruses were utilized to transduce neurosphere cultures in a BSL2 facility. The cells were incubated with the indicated virions and transgene expression, or their downstream effects, were assessed at least 72 h post-transduction.

## 3. Results

### 3.1. NGF Neutralization Promotes Oligodendrogenesis and miR-219 Up-Regulation in Vivo

**Figure 1:**
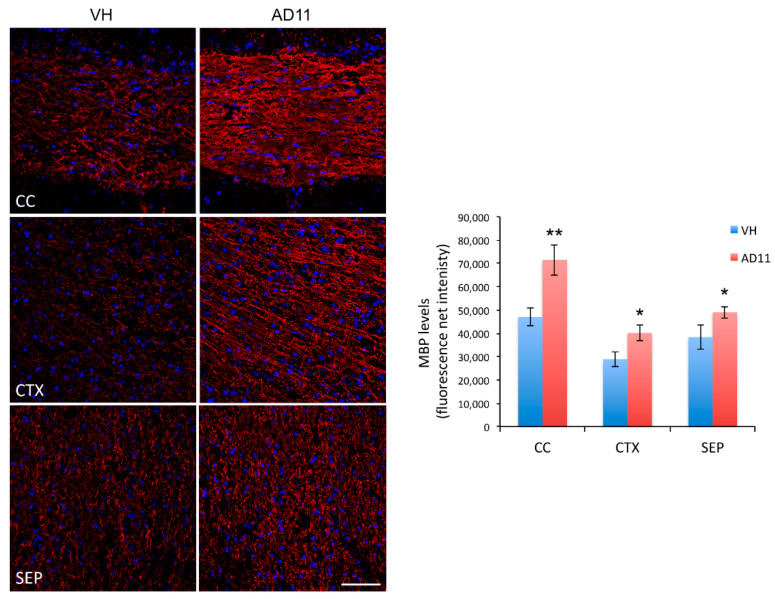
NGF neutralization increases myelination in vivo. Anti-myelin basic protein (MBP) immunostaining (in red) is higher in AD11 brain areas such as the corpus callosum (CC), the cerebral cortex (CTX), and the septum (SEP), as compared to control animals (VH). The histogram reports the quantification of the net fluorescence intensity in these areas. Nuclei labeled with 4,6-diamidine- 2-phenylindole dihydrochloride (DAPI) are in blue. Scale bar 75 µm; magnification 40×. The results are expressed as the mean ± standard error (SEM) from three animals for each genotype (quantifying six slices per animal). Statistical significance is calculated relative to the VH sample for each area. Student’s *t*-test: * *p* < 0.05, ** *p* < 0.01.

To investigate the link between the NGF system and myelination, we analyzed the expression of MBP in the brain of AD11 mice, a transgenic line in which expression of the anti-NGF αD11 antibody leads to a persistent postnatal neutralization of mature NGF. Strikingly, AD11 mice showed enhanced immunoreactivity for MBP in different areas, including cerebral cortex (CTX), corpus callosum (CC), and septum (SEP) (Figure 1).

**Figure 2:**
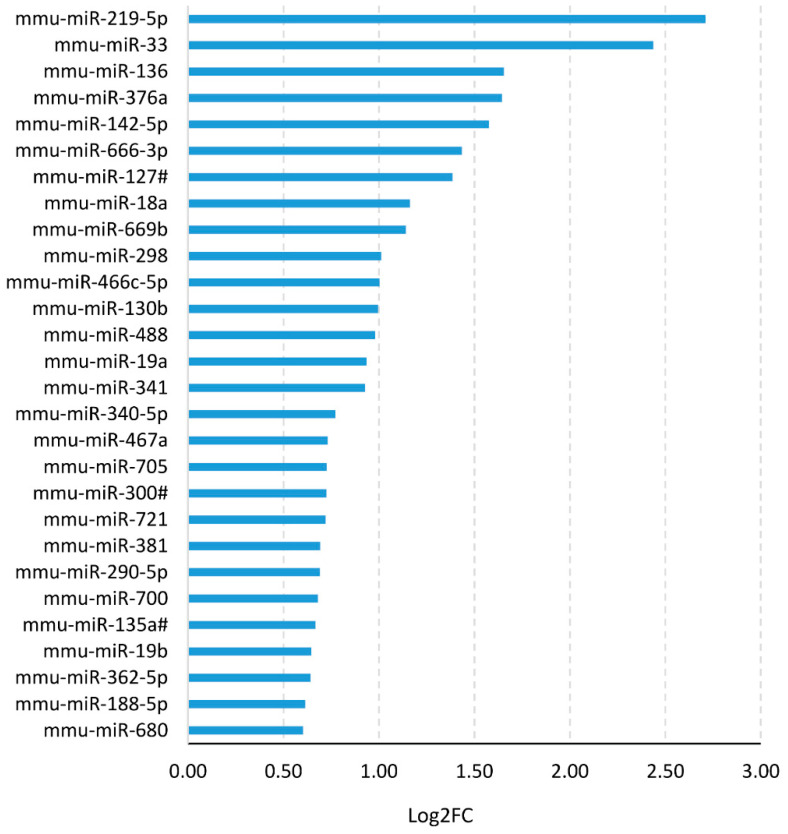
MicroRNA-219 (MiR-219) is up-regulated in AD11 mice. Relative Log2 expression ratio for a selected list of differentially expressed microRNAs in the hippocampus of AD11 anti-NGF mice at one month of age (AD11 vs. VH control, *n* = 4 vs. *n* = 4 samples). The list includes miR-219, which shows the largest fold change ratio. MicroRNA genes have been selected from a microarray experiment, using the following criteria: |Log2 FC| > 0.58 (FC > 1.5 in linear scale), heteroscedastic Student’s *t*-test *p* < 0.05.

This result strongly suggests that NGF neutralization promotes in vivo myelination, which is in agreement with its previously reported inhibitory effect on OL differentiation [17,19,20]. Since microRNAs (miRNAs) are emerging as key regulators of OL differentiation, we performed a microRNA expression profile by microarray analysis of RNA extracted from AD11 mouse hippocampi at one month of age. Among the microRNAs whose expression was significantly altered (Figure 2), we focused on miR-219 due to its strong up-regulation and its known involvement in oligodendrogenesis [41].

**Figure 3:**
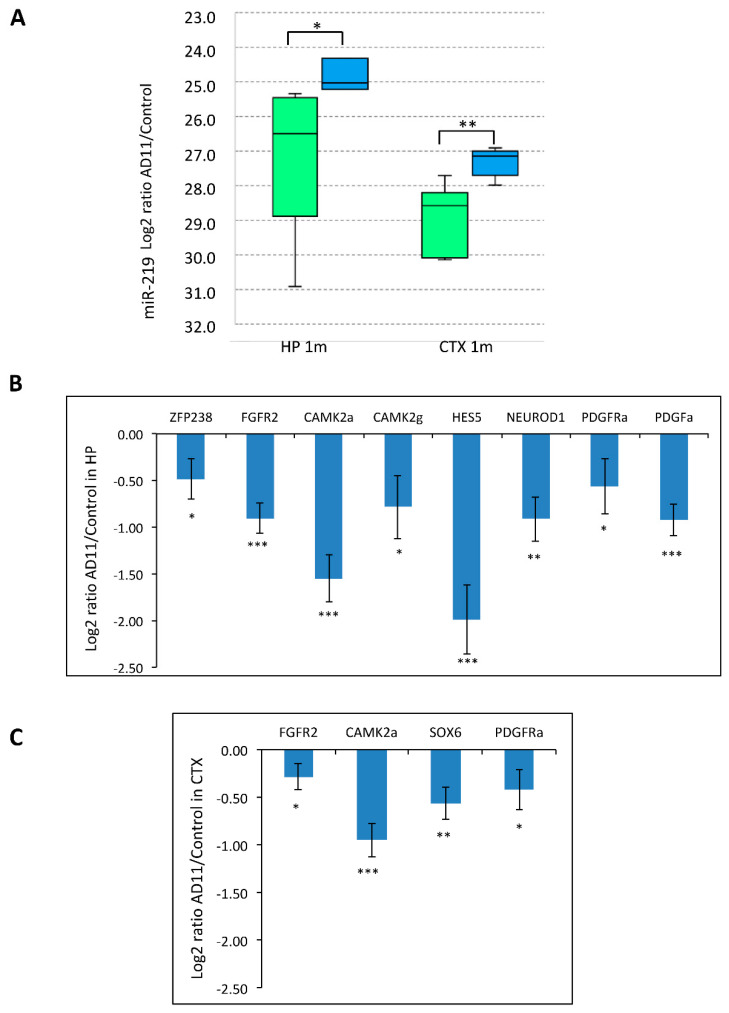
Validated miR-219 target mRNAs are downregulated in AD11 brains. (**A**): Up-regulation of miR-219 in the cortex (CTX) and hippocampus (HP) of AD11 mice at 1 month of age, by qRT-PCR. The normalized Ct of AD11 mice (blue) and VH mice (green) are shown. Statistical significance by one-tail heteroscedastic Student’s *t*-test: * *p* < 0.05, ** *p* < 0.01. The Ct axis is in reverse order to highlight the up-regulation of miR-219. The results are expressed as the mean ± standard error (SEM) from independent experiments (*n* = 3–7). (**B**,**C**): Log2 ratio of differentially expressed miR-219 target mRNAs in AD11 hippocampus (**B**) and cortex (**C**) at one month of age by qRT-PCR (AD11/VH control). The results are expressed as the mean ± standard error (SEM) from independent experiments (*n* =7). Statistical significance is calculated relative to the control samples. Student’s *t*-test: * *p* < 0.05, ** *p* < 0.01, *** *p* < 0.001.

We confirmed its up-regulation in AD11 hippocampi (hp) by Real-Time qRT-PCR and showed that miR-219 is also strongly up-regulated in the cortex (CTX) (Figure 3A). Consistently, we also verified that the known miR-219 target mRNAs were down-regulated in the same brain areas (Figure 3B,C).

**Figure 4:**
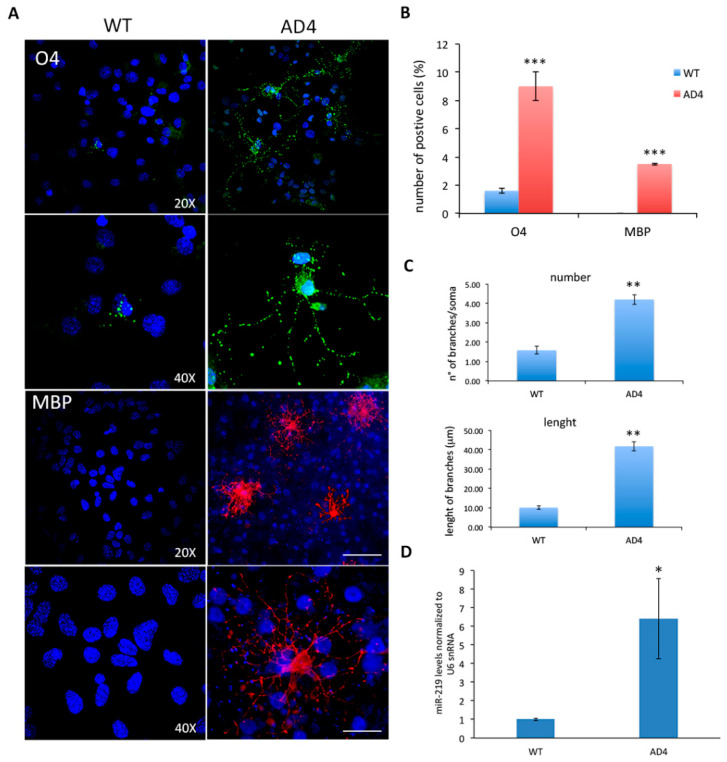
NGF neutralization promotes oligodendrogenesis in AD4 adult hippocampal progenitors. (**A**): Immunostaining for O4 (green) and MBP (red) in AD4 and in wild type (WT) neurosphere cultures upon oligodendrocyte (OL) differentiation. (**B**): Quantification of O4+ cells as in **A** (a total of 592 AD4 cells and 924 WT cells were examined). (**C**): AD4-derived OLs are more differentiated, in terms of number and length of branches and in terms of MBP expression, when compared to WT-OLs, as quantified in the histograms. Nuclei labeled with DAPI are in blue. Scale bars, 50 and 10 µm, magnification 20× and 40×, respectively. (*n* = 20). (**D**): Up-regulation of miR-219 in AD4 neurospheres by qRT-PCR from four independent experiments (*n* = 4). The results are expressed as the mean ± standard error (SEM). Fold change and Statistical significance are calculated relative to the WT sample. Student’s *t*-test: * *p* < 0.05, ** *p* < 0.01, *** *p* < 0.001.

Among them, we found platelet growth factor receptor alpha (PDGFaR), SRY-box-containing gene 6 (SOX6), and zinc finger protein 238 (ZFP238), all being involved in promoting OPC proliferation and inhibiting OL differentiation [32,45,46]. These data suggest that NGF neutralization increases myelination by promoting oligodendrogenesis through miR-219 up-regulation. To test this hypothesis, we utilized in vitro cultures of neurospheres as paradigm of OL differentiation in a context of NGF deprivation. Neurosphere cultures are known to contain OPCs that can be expanded and differentiated into oligodendrocytes in vitro [43]. NGF-deprived neurospheres, namely AD4 [41], derived from AD11 mice hippocampi. Upon differentiation of AD4 and WT neurospheres towards the OL lineage (see Materials and Methods), we performed immunofluorescence staining for the oligodendrocyte markers O4 and MBP. We found that AD4 neurospheres give rise to more O4+ and MBP+ oligodendrocytes (O4: 9% ± 1%; MBP: 3.5% ± 0.07%) than WT cells (1.6% ± 0.2%; MBP: 0%) (Figure 4A,B). Strikingly, while we could observe MBP staining in 3.5% of AD4-derived OLs (± 1%), no MBP+ OLs were detected in WT samples and AD4-derived OLs were also more differentiated in terms of number and length of processes extending from the soma (Figure 4C). The enhanced differentiation of AD4-OLs, compared to WT cells, was accompanied by increased miR-219 expression in AD4 neurospheres (Figure 4D), suggesting a direct link between NGF neutralization, miR-219 up-regulation, and oligodendrogenesis.

### 3.2. Oligodendrogenesis in AD4 Neurosphere Is Dependent on miR-219

**Figure 5:**
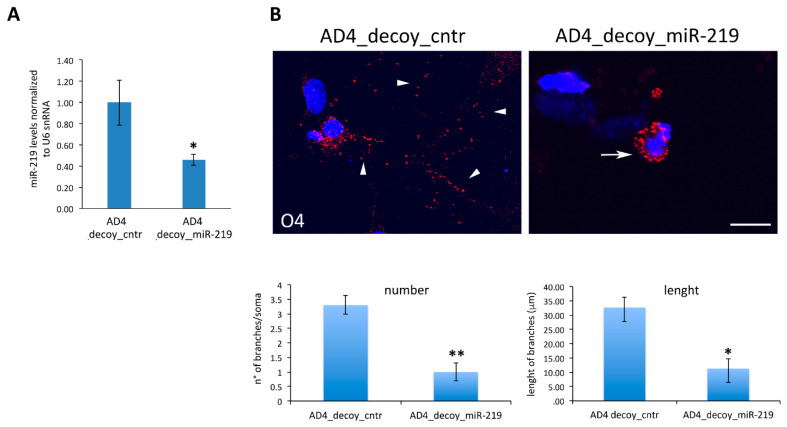
NGF neutralization promotes OLs differentiation through miR-219 modulation. (**A**): MiR-219 down-regulation in AD4 neurospheres upon transduction with a decoy_miR-219 lentivirus. qRT-PCR results are reported as the ratio between miR-219 levels in decoy_miR-219 versus decoy_miR_cntr infected AD4 cells. The results are expressed as the mean ± standard error (SEM) from five independent experiments (*n* = 5). (**B**): Immunostaining for O4 (red) in AD4 progenitors infected with decoy_cntr or decoy_miR-219 lentiviruses. Inhibition of miR-219 in AD4 cells leads to poorly differentiated OL, without processes (arrow), compared to decoy_cntr infected cells (arrowheads), as quantified in the histograms (lower panel) (*n* = 20 O4+ cells). Scale bar 10 µm, magnification 60×. The nuclei labeled with DAPI are in blue. Statistical significance is calculated relative to the AD4 decoy_cntr sample. Student’s *t*-test: * *p* < 0.05, ** *p* < 0.01.

To evaluate whether the enhancement in oligodendrogenesis triggered by NGF deprivation is dependent on the up-regulation of miR-219 we exogenously depleted miR-219 in AD4 neurospheres. To this end, we took advantage of a lentivirus over-expressing an anti-miR-219 tough decoy inhibitor (decoy_miR-219), which effectively down-regulates miR-219 levels (Figure 5A). AD4 neurospheres transduced with the miR-219 decoy lentivirus, or with a control non-targeting lentivirus (decoy_cntr), were induced to differentiate and the degree of OL differentiation was analyzed by immunofluorescence assays. The results, as shown in Figure 5B, revealed that the lentiviral-mediated inhibition of miR-219 severely counteracts the increase in OL differentiation observed upon NGF deprivation, as also assessed by analyzing the degree of O4+ arborization and the percentage of MBP+ cells in miR-219 decoy cultures versus control cultures (Figure S1).

**Figure 6:**
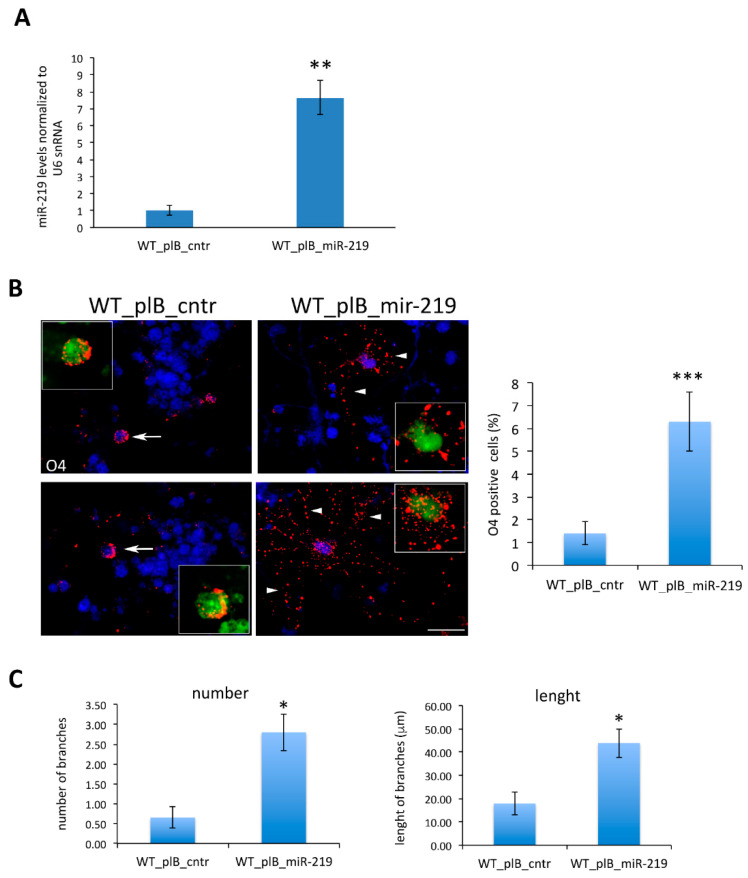
miR-219 overexpression promotes OL differentiation of WT neurospheres. (**A**): MiR-219 up-regulation in WT neurospheres driven by transduction with the plB_miR-219 lentivirus. Real-Time qRT-PCR results are expressed as fold-change of miR-219 levels in WT cells transduced with plB_miR-219 versus plB_cntr lentiviruses. Data are reported as the mean ± standard error (SEM) from three independent experiments (*n* = 3). (**B**,**C**): MiR-219 overexpression increases the number of O4+ cells and their differentiation. WT neurospheres were transduced with plB_cntr or plB_miR-219 lentiviruses (GFP positive) and differentiated in vitro. Data are expressed as the mean ± standard error (SEM) (**B**) Right panel: representative images of O4 immunostaining (in red) and DAPI (in blue). Scale bar 20 µm, 40× magnification. The inset in each panel represents a 1.5 magnification of transduced GFP-positive cells. Branch length is highlighted with arrows (short branches) or arrowheads (long branches). Right panel; histogram reporting the percentage of O4+ cells in transduced WT cultures (WT_plB_cntr *n* = 573; WT_plB_miR-219 *n* = 423). (**C**) Histograms reporting the number and length of O4+ branches in transduced WT cultures (*n* ≥ 20 O4+ cells for each condition). (**A**–**C**) Statistical significance is calculated relative to the WT_plB_cntr sample. Student’s *t*-test: * *p* < 0.05, ** *p* < 0.01, *** *p* < 0.001.

This result indicates that NGF neutralization requires an up-regulation in miR-219 levels to promote oligodendrogenesis. As a complementary approach, we next tested whether overexpression in WT neurospheres of miR-219 was alone sufficient for driving an increase in oligodendrogenesis that was similar to that observed upon NGF deprivation. To this end, we infected WT neurospheres with a lentivirus driving the expression of miR-219 and the Green Fluorescent Protein (pLB_miR-219), or with a control lentivirus encoding a non-targeting miRNA (pLB_cntr). In agreement with the known role of miR-219 in oligodendrogenesis, its overexpression in WT neurosphere cultures (Figure 6A) was indeed sufficient to drive an increase in O4+ cells (Figure 6B). Moreover, miR-219 overexpressing cells show longer branches than those that were observed in control-infected cells (Figure 6C). Strikingly, both WT and miR-219-overexpressing WT neurospheres fail to fully mature into MBP-expressing OLs, as observed in AD4 neurosphere cultures (Figure 4 and Figure S1). This observation suggests that NGF deprivation promotes oligodendrogenesis via other pathways in addition to miR-219 up-regulation.

### 3.3. Modulation of miR-219 Expression Is Dependent on NGF

**Figure 7:**
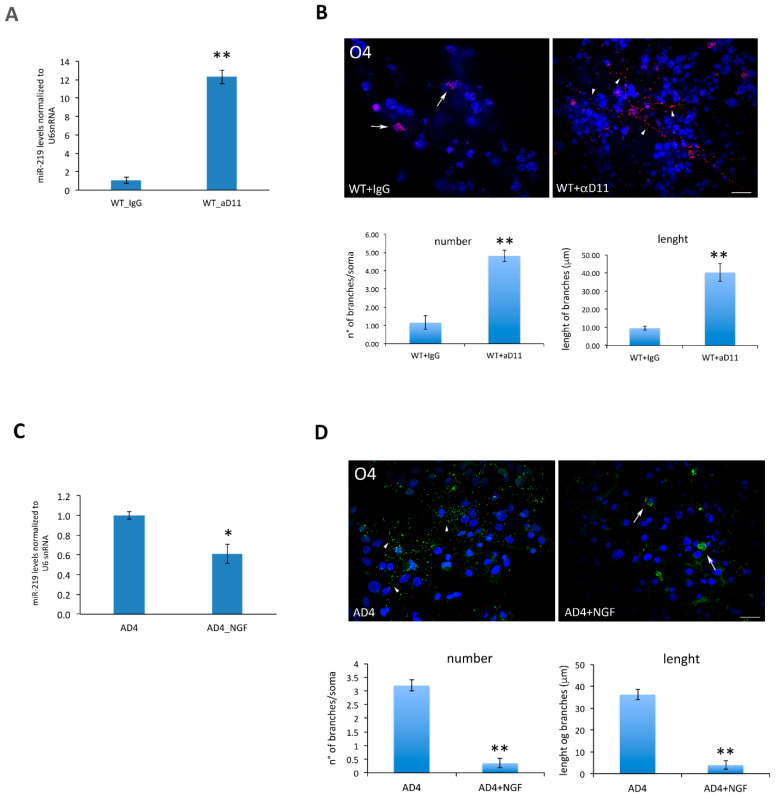
Modulation of miR-219 is NGF-dependent. (**A**) NGF neutralization in WT cells, by treatment with the anti-NGF αD11 antibody, induces a significant increase in miR-219 levels, which is not observed in control IgG-treated cells. The histogram reports qRT-PCR results of miR-219 levels measured in WT cells treated with αD11 or IgG as control (*n* = 3). (**B**): Representative immunofluorescence images for O4, showing enhanced differentiation of anti-NGF-treated WT oligodendrocytes (αD11) compared to IgG-treated WT oligodendrocytes, in terms of number and length of branches, as quantified in the histograms (lower panel). Nuclei labeled with DAPI are in blue. Scale bar, 10 µm, 20× magnification. (**A**,**B**) Statistical significance is calculated relative to the WT + IgG sample. Data are expressed as the mean ± standard error (SEM) (**C**): NGF treatment (45 min. long) of AD4 progenitors induces a down-regulation of miR-219 levels. qRT-PCR results are reported as ratio between miR-219 levels measured in treated versus untreated AD4 cells (*n* = 4). (**D**): Chronic NGF treatment reduces OL differentiation. Immunofluorescence for O4 shows less differentiated OL in NGF-treated AD4 cultures, compared to untreated cells. Scale bar, 10 µm, 20× magnification. The results were quantified (lower panel) both in terms of branch number and length (*n*≥ 20 O4+ cells for each condition). (**C**,**D**) Statistical significance is calculated relative to the AD4 untreated sample. The data are expressed as the mean ± standard error (SEM)). Student’s *t*-test: * *p* < 0.05, ** *p* < 0.01. (**B**,**D**). Branch length is highlighted with arrows (short branches) or arrowheads (long branches).

To further assess the causal link between NGF deprivation, miR-219 up-regulation, and oligodendrogenesis, we modulated NGF levels in WT and AD4 neurospheres prior to OL differentiation. Specifically, we treated WT cells with anti-NGF αD11 antibody at a concentration that mainly neutralizes the mature form of NGF (100 ng/ML) [41], while AD4 neurospheres were treated with NGF at 50 ng/mL, a concentration that is required to counteract the neutralizing action of the endogenous αD11 antibody [43]. Upon five days of αD11 treatment, miR-219 expression significantly increased in WT neurospheres as compared to the control IgG-treated cells (Figure 7A). As a result, αD11-treated O4+ cells showed a greater branching of their processes (Figure 7B). On the other hand, NGF-treated AD4 neurospheres expressed lower levels of miR-219 (Figure 7C), and generated poorly differentiated OLs, devoid of processes extending from the cell body, when compared to the mature O4+ oligodendrocytes that were obtained from AD4 OPCs (Figure 7D).

### 3.4. Anti-NGF αD11 Neutralization of NGF Increases OL Differentiation of Rat Primary OPCs

**Figure 8:**
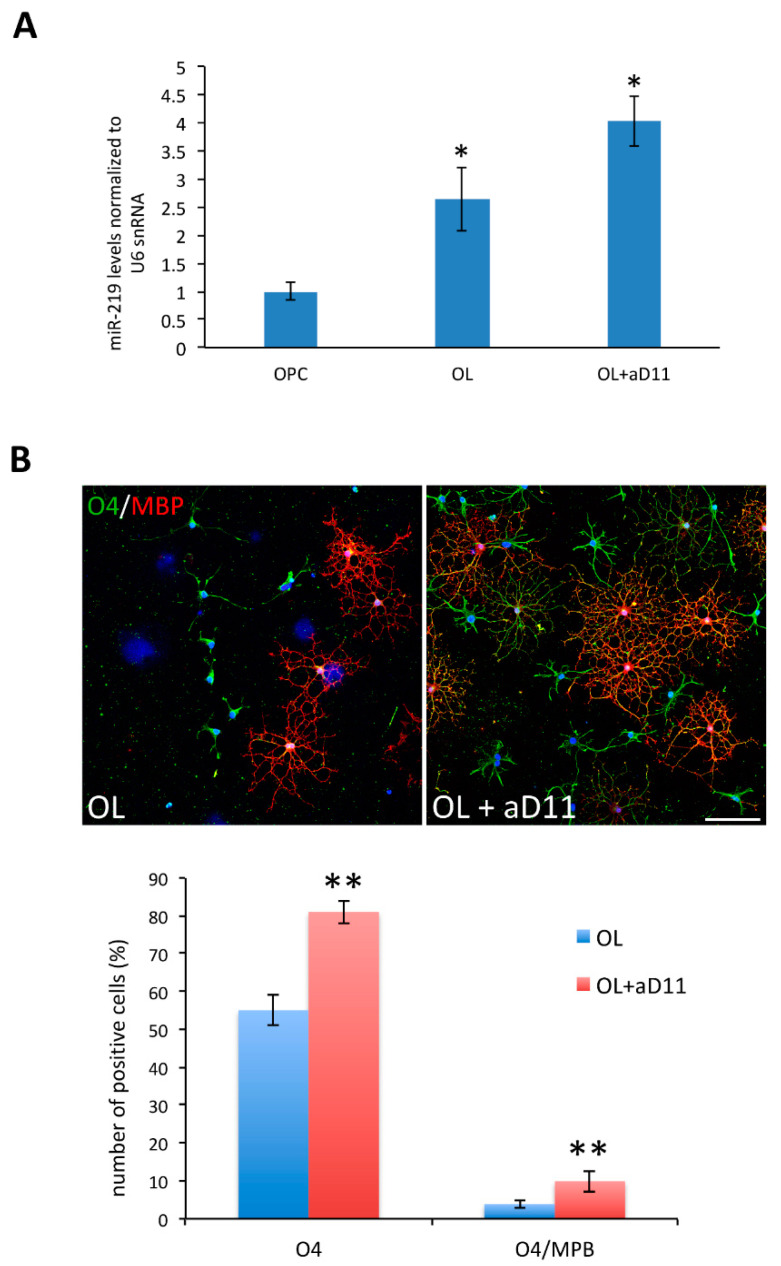
NGF neutralization increases OLs differentiation of primary OPC cultures. (**A**): miR-219 up-regulation by qRT-PCR in rat OPC cultures upon in vitro differentiation and αD11 antibody treatment (100 ng/mL), compared to IgG-treated cells. Statistical significance is calculated relative to the OPC undifferentiated sample. Data are expressed as the mean ± standard error (SEM) (*n* = 3). (**B**): immunostaining for MBP (red) and O4 (green) of rat OPC cultures upon in vitro differentiation. αD11 antibody treatment gives rise to more O4+ pre_OL (green cells) and O4 + MBP+ mature OLs (green and red cells), as quantified in the histogram (lower panel). Nuclei labeled with DAPI are in blue. Scale bar 10 µm, 40× magnification. Statistical significance is calculated relative to the untreated sample. The data are expressed as the mean ± standard error (SEM) (OL *n* =252; OL + αD11 *n* = 186). Student’s *t*-test: * *p* < 0.05, ** *p* < 0.01.

In order to test NGF neutralization as a viable strategy to promote oligodendrogenesis and myelination in a different cellular context, we treated rat primary OPCs with anti-NGF αD11 antibody. These primary cultures represent a well-established in vitro model of OL differentiation, in which the key role played by miR-219 has been previously demonstrated [32]. Interestingly, anti-NGF αD11 (100 ng/mL) treatment of rat OPCs during OL differentiation further increased miR-219 expression in OLs as compared to OPCs (Figure 8A). Additionally, the number of O4+ pre-oligodendrocytes and of O4+ MBP+ mature OLs derived from αD11-treated OPC primary cultures was significantly higher (Figure 8B) than in control cultures. Altogether, our results demonstrate a new role for NGF in regulating adult oligodendrogenesis and myelination through the modulation of miR-219 levels.

## 4. Discussion

MiR-219 is the most highly expressed miRNA in OLs [33], acting as a key switch for the differentiation of mouse OPCs into mature myelinating OLs [32]. Reduced levels of miR-219 have been observed in the cerebrospinal fluid of Multiple Sclerosis (MS) patients [31] and may contribute to impaired OPC differentiation and failed remyelination [32,34]. Conversely, increasing the miR-219 levels leads to enhanced OPC maturation and it could serve as a viable strategy towards remyelination [47,48,49,50,51,52]. Overall, miR-219 is an established enhancer of oligodendrogenesis and a promising target for remyelination. However, very little is known of the endogenous upstream signaling pathways that modulate this key effector during oligodendrogenesis.

In this study, we revealed that NGF neutralization promotes oligodendrogenesis by increasing the expression of miR-219. We provide evidence of this novel interplay both in vivo, in a murine model of NGF neutralization (AD11 mice), and in vitro, in NGF-deprived neurospheres and primary rat OPCs. In either system, NGF neutralization promotes myelination by increasing miR-219 and, consequently, the number of O4+ pre-OLs and mature MBP+ OLs. These findings provide a mechanistic explanation to the previously reported inhibitory role of NGF on OL differentiation [17,19,20]. In particular, NGF was shown to induce the expression of axonal LINGO-1 [19], a component of the NgR1 (Lingo)/p75^NTR^ signaling complex that negatively regulates myelination by OL [53]. This evidence entails that NGF indirectly affects oligodendrogenesis, modulating regulatory axonal signals. However, our results in OPC cultures suggest that NGF can also inhibit oligodendrogenesis in a cell autonomous fashion, independently of neuronal signals. Hence, this study unveils an additional and parallel pathway by which NGF participates in the inhibition of myelination, directly acting on oligodendrocytes. Further, we show that the molecular pathway linking NGF signaling to oligodendrogenesis requires the modulation of miR-219 expression, an established enhancer of OPC differentiation. Indeed, in NGF-deprived brains, miR-219 up-regulation results in the down-regulation of target genes that are involved in modulating OPC differentiation directly, such as PDGFaR, SOX6, and ZFP238. Interestingly, LINGO-1 mRNA was also recently found to be a target of miR-219 [34]. This evidence, together with our data, suggests that NGF may control both cell-autonomous and non-autonomous pathways, which converge to curtail oligodendrogenesis, via the inhibition of miR-219. In neurons, NGF would lead to increased LINGO inhibitory signaling from axons, while, in OPC, it would result in up-regulation of miR-219 target genes that sustain OPCs proliferation. Further studies will be required to test this scenario and address the contribution of each of these NGF-controlled pathways towards oligodendrogenesis.

The finding that NGF modulates myelination via miR-219 hints at a potential involvement of NGF in myelin plasticity. Myelination in the CNS is dynamically modulated by neuronal activity, even in a neuron-specific fashion [54], and it contributes to brain plasticity, learning, and memory [55]. Consistently, mir-219 is strongly upregulated in the visual cortex at the peak of the critical period, with respect to its onset [56]. Given the known, yet unexplained, role of NGF in regulating visual cortical plasticity [57], it is tempting to speculate that an activity dependent modulation of oligodendrogenesis by NGF might be involved in the regulation of visual cortical plasticity and the critical period.

In addition to unveiling the molecular mechanism linking NGF to oligodendrogenesis, our study may also have therapeutic implications for demyelinating diseases, such as MS [58]. Indeed, reduced levels of miR-219 have been measured in MS patients [31], leading to impaired OPC differentiation and failed remyelination [32,34]. On the other hand, the intranasal delivery of miR-219-enriched exosomes promotes myelination in aged rats [52] and in EAE models of MS [51], while nanofiber-mediated miR-219 delivery enhances the differentiation and maturation of OPCs [47]. Thus, increasing the miR-219 levels could be a viable strategy towards remyelination. In this view, understanding the molecular cascade leading from NGF signaling to miR-219 will pose the basis for developing new therapeutic strategies. For example, novel downstream NGF targets could be identified and exploited for modulating miR-219 without affecting the NGF system homeostasis as a whole.

Notably, NGF is also a key element in the neuro-immune network and it is induced in inflammatory processes [59], including MS [20,60,61]. The finding that increased levels of NGF were detected in cerebrospinal fluid and brain of both MS patients and EAE mice [60] suggests that NGF may participate in linking inflammation to demyelination. However, these analyses did not take the immature precursor of NGF (named proNGF) into account, since the available antibody measurements do not discriminate between NGF and proNGF [62]. Thus, a full characterization of the role that is played by the entire NGF system in OL ontogenesis deserves further investigation, including a detailed analysis of the combinatorial expression of proNGF, NGF and of their receptors (p75NTR, TrkA and sortilin) during physiological OL maturation and in the context of demyelinating pathological conditions. In this view, single-cell RNA-sequencing studies, with a focus on oligodendrocyte differentiation, would be a valuable approach to identify at which stage of oligodendrogenesis NGF is acting. To this end, we performed a preliminary analysis of two transcriptomic datasets [63,64] that interrogate mouse and human oligodendrogenesis in physiological and MS conditions. This analysis did not reveal a clear stage-specific modulation of TrkA nor of p75NTR. However, a more exhaustive investigation is required in order to conclusively ascertain potential variations in NGF receptor levels at different stages of oligodendrocyte differentiation.

Our data show that miR-219 up-regulation is necessary for the observed enhancement of oligodendrogenesis that is elicited by NGF deprivation. However, increasing the miR-219 levels in the WT neurospheres alone is not sufficient for driving the same degree of OL maturation (specifically MBP expression), as observed in AD4 neurospheres. This suggests that NGF deprivation elicits oligodendrogenesis via multiple pathways, not exclusively through miR-219 up-regulation. Elucidating these additional NGF-controlled pathways is a necessary step towards a more exhaustive understanding of the role played by NGF in myelin formation. Additionally, further characterization of NGF-signaling downstream effectors could lead to the identification of additional therapeutic targets to be exploited, in synergy with miR-219, in the treatment of demyelinating diseases.

This study has some limitations, as often occurs in research. As reported above, we did not analyze the entire NGF system or characterize the downstream signaling pathway leading from NGF deprivation to mir-219 up-regulation. Moreover, as source of chronically NGF-deprived OPCs, we used adult neurospheres, which have the advantage of reproducing the entire process of OL ontogenesis in vitro, from early pre-progenitors (OPP) to myelinating OLs. However, we did not characterize the specific differentiation stage during which NGF action is required to control OL maturation. Obtaining this information would entail additional cell biology experiments, such as a detailed analysis of both NGF and its receptors expression, in combination with OPP/OPC/OL specific markers, during each step of OL differentiation. Alternatively, time-course experiments in which NGF signaling is inhibited at different phases of OL maturation could also be instructive. Finally, the use of genetic mouse models for NGF receptors (p75 or TrkA null mice) might help to better investigate the role of NGF and its signaling pathways during oligodendrogenesis.

## Data Availability

The data presented in this study are openly available and can be found here: [https://www.ncbi.nlm.nih.gov/geo/query/acc.cgi?acc=GSE162675] (accessed on 17 February 2021).

## Supplementary Materials

The following are available online at https://www.mdpi.com/2073-4409/10/2/405/s1, Figure S1: NGF neutralization increases OLs differentiation via additional pathways besides miR-219 downregulation.
