# Supplementary figures and images for: Nerve Growth Factor Neutralization Promotes Oligodendrogenesis by Increasing miR-219a-5p Levels
Source: Cells. 2021 Feb 16;10(2):405. doi: 10.3390/cells10020405 (PMC7920049; doi:10.3390/cells10020405)

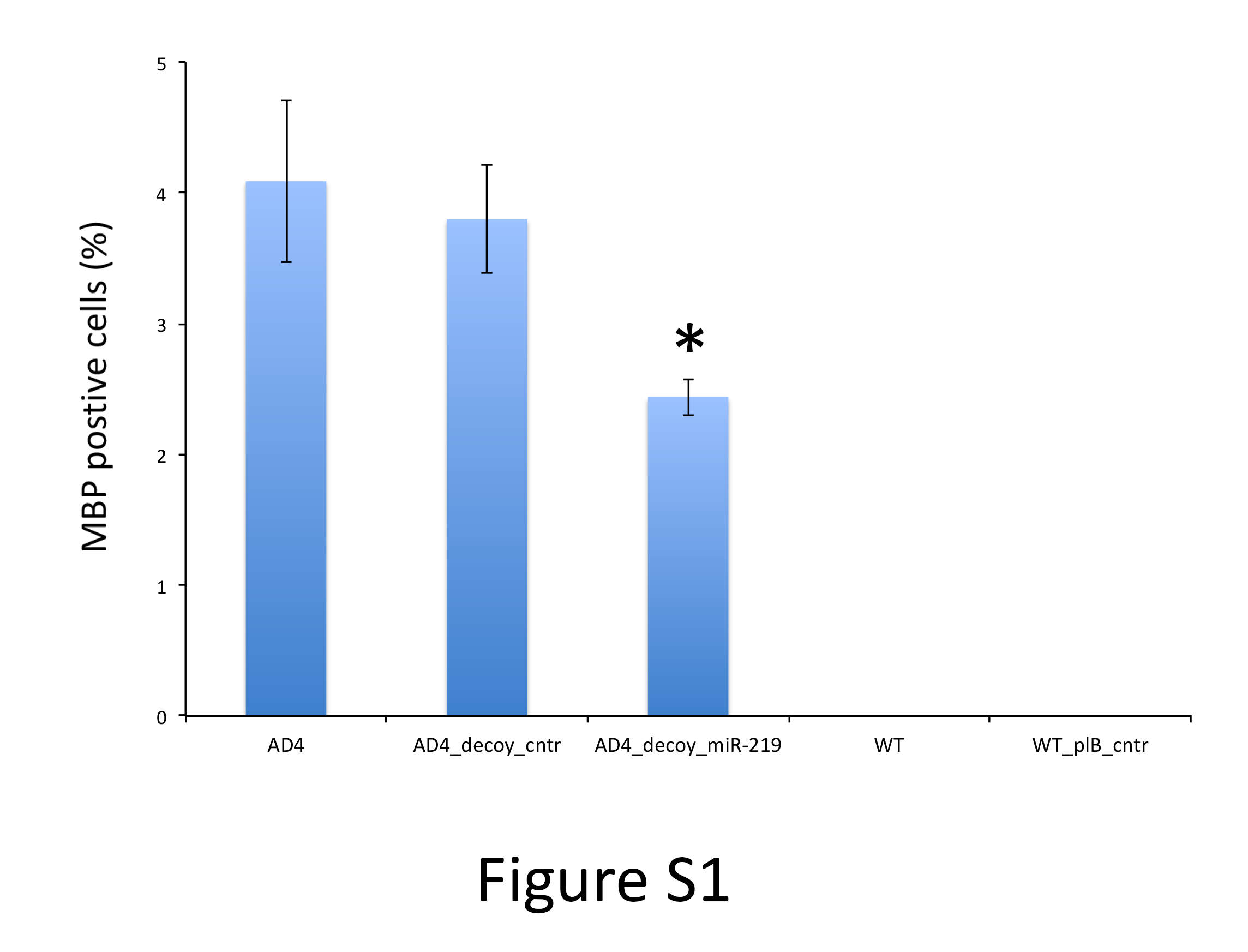

Supplement: Supplementary file 1 [file cells-10-00405-s001.zip › cells-1083668-supplementary.tif]
